# Assessment of the Quality of Obstetric Services From the Perspective of Maternity Patients and Service Providers in a Tertiary Care Obstetric Unit in Lithuania

**DOI:** 10.1177/11786329231180790

**Published:** 2023-06-24

**Authors:** Justina Katinaitė-Vaitkevičienė, Aleksandras Patapas

**Affiliations:** 1 Mykolas Romeris University, Vilnius, Lithuania; 2 Vilnius University, Vilnius, Lithuania

**Keywords:** Healthcare service quality, patient, medical staff, midwifery, quality assessment

## Abstract

Although largely focused on the patient, the provision of healthcare services is a 2-way process and its success hinges on the interactions between patients and physicians. Given the growing role of subjective, patient-dependent assessment of the quality of care received, which is increasingly influenced by the individual aspects of the interaction between patients and care providers, in addition to the explicitly measurable, objective assessment of the quality of care received based on clinical indicators, quality assessment of services should especially consider and explore the attitudes, needs and dynamics of all the parties involved in the healthcare process. This study was designed to assess the attitudes of maternity patients and healthcare providers towards the quality of obstetric care. A quantitative questionnaire survey was conducted in a tertiary level healthcare facility providing obstetric services in Lithuania. Research findings suggested that maternity patients rate both the technical and functional quality of obstetric services higher than the staff providing it. Midwives and obstetricians-gynaecologists view quality assurance as a complex process, rather than focus solely on quantitative indicators. Since midwives were rated slightly higher than physicians in terms of services they provide, it may be appropriate to ensure and encourage a wider use of midwife-only deliveries in low-risk births. A comprehensive assessment of the quality assurance aspects as viewed by the patients and the staff should be included in the regular quality assessments of healthcare facilities as one of the most informative assessment tools on the service quality.

## Introduction

Midwifery is one of the medical disciplines that is continuously undergoing changes both in terms of technical quality (the external expression of quality relating to the material and service delivery tools, technological process of service delivery) and functional quality (relating to the manner in which the service is delivered and the context in which it is delivered). The clinical practice guidelines used in Lithuanian maternity units are reviewed and updated every few years based on global recommendations and represent some of the best prepared guidelines available to healthcare professionals, adapted to the national profile. Recently, there also have been some important changes in obstetrical field in Lithuania as caesarean section (C-section) at the request of the pregnant woman became an option for delivery with psychological evaluation prior to final decision of choosing c-section as preferable childbirth method. In such cases all services involving childbirth care are chargeable^1^ although in Lithuania all healthcare services provided for pregnant women regarding pregnancy care and childbirth care are free of charge, financed by state compulsory health insurance.

Rapid change has also been observed globally in terms of functional quality, with the emergence of new concepts specific to midwifery and maternity patient care, such as Respectful Maternity Care (RMC), as defined by the World Health Organization in 2018, to improve the delivery of services.^2^ This dynamic pace of midwifery is shaping the need to investigate and analyse changes in patient satisfaction with the quality of services, both in terms of the well-established aspects of service quality from the patient’s perspective (information, confidentiality, safety) and the more recently highlighted aspects of service satisfaction (empowerment in decision-making, empathic attitude of staff towards the patient, effective communication).^2,3^

In studies assessing service quality from patient’s perspective, both researchers and patients tend to focus on the functional quality aspects, with communication difficulties in interactions with medical staff being the most important aspect of patient satisfaction and the most problematic at the same time.^4,5^ This tendency is widely observed in obstetrics, as well as in other fields of medicine. Important to mention that the nature of midwifery itself is the reason for the need to investigate communication aspects in more detail because it is crucial that the maternity patient communicates successfully not only with the doctor, but also with the midwife. It should be noted that in most countries, the presence of a midwife alone in low-risk births is becoming a common obstetric practice. As a direct part of the doctor-patient (or midwife-patient) relationship, the aspect of information provision to the patient is integral to the assessment of communication and maternity patients associate good communication with fewer staff and the opportunity to be involved in the decision-making throughout their care.^4^ This only proves that childbirth is a very intimate moment in a woman’s life. The absence of a doctor should not be distressing for women if they are able to establish a good relationship with the midwife attending the birth, while the service quality may be rated even higher than if the whole obstetric team was present. Meanwhile in Lithuania at this time, typically, a whole team of professionals is involved in obstetrical care including midwives and obstetricians-gynaecologists regardless of whether it is low or high-risk childbirth, anaesthesiologists and neonatologists joining the medical team depending on clinical situation. The level of interaction between midwives and obstetricians is high with decision-making often left only for doctors so midwives have low level of autonomy which basically contradicts the statements found in midwifery medical standard established by the government.^6^

Although there are numerous studies examining the quality of services from the patient’s perspective, there are few studies comparing doctors’ and patients’ perceptions of the quality of care provided, and there are almost no studies of this kind in midwifery, especially in Lithuania. Aniulienė et al,^7^ in their study of the quality of outpatient obstetric services, concluded that patients were statistically more likely to rate the technical quality of the services higher, whereas the functional quality of care was rated similarly by both the staff and the patients.^7^

Another worrying aspect of healthcare is that experts acknowledge that medical training programmes focus on biomedical care missing out humanistic aspects of care.^8^ Likewise, in Lithuania, the training programmes for medical residents are currently focused exclusively on biomedical care, while the development of interpersonal skills is limited to the direct contact with patients in the work environment. Potential communication difficulties among staff should also be considered in terms of their influence on the quality of care provided. In Brazil, which has the highest rate of C-section operations (as many as 52% of deliveries are completed surgically), a study found that there are persistent difficulties in coordinating work flows, there are differences in the attitudes of physicians and midwives towards the factors determining the quality of care, which in turn negatively affects the ability to work in teams.^9^ To some extent, teamwork challenges can also be observed in Lithuanian obstetric units. The technical focus of physicians, the use of medications to modify the natural course of labour, as well as the need for and performance of instrumental interventions often conflict with the idea of a more natural childbirth promoted by the majority of midwives. This is rather a universal phenomenon, independent of cultural or other differences worldwide but noticeably there are no researches conducted on the level of satisfaction with interpersonal interactions between midwives ant doctors in Lithuania. Obviously, such researches would be welcomed and contribute to better understanding on the need of changes regarding better service provision for patients which is highly related to professional and interpersonal relationships in certain work environment.

Although the midwife alone may be involved in a low-risk birth, each obstetric case is, of course, individual. Therefore, it is always necessary to be mindful of the dynamic nature of the obstetric situation, where a low-risk birth can develop complications in a matter of minutes. Only the concerted and timely efforts of the staff as a team can ensure a positive outcome for the mother and the newborn. More research is clearly needed on the specificities of teamwork, given its importance for the outcome of care.

In summary, no studies on the satisfaction with the quality of midwifery services are conducted nationally, and the only officially available data are those reflecting the technical quality of care, such as the number of deliveries, the incidence of C-sections and the data related to birth outcomes. Patient satisfaction often remains the subject of research by individual researchers. In obstetric care, technical and functional quality are equally important, as women tend to evaluate the quality of care as a whole.^10^ In economically developed countries, technical quality is generally rated well, but women’s dissatisfaction with the functional quality of care is linked to the quality of information provision, interpersonal communication with the care provider and the degree of their participation in the decision-making process during the delivery process. Since the success of the childbirth depends directly on the concerted efforts of the obstetric team, which usually includes obstetricians-gynaecologists, midwives, neonatologists and anaesthetists as well as the effective collaboration of this team with the mother, it is essential to consider the needs of the staff and the challenges they face in the working environment to ensure the quality of the service.

Research objective – to assess the attitudes of maternity patients and medical staff (physicians, midwives) towards the quality of obstetric care provided in a tertiary care obstetric unit.

## Research Methodology

### Research organisation

A quantitative study using a questionnaire survey method was conducted in a tertiary care obstetric unit with highest number of births in the country per year.^11^ Important to mention that in tertiary level maternity units in Lithuania services being provided in cases of low-risk pregnancies as well as high-risk pregnancies (in contrary to secondary level obstetric units where mainly only care for low-risk childbirths can be provided) with high-risk pregnancies and childbirths outnumbering those of low-risk.^12^ A total of 364 completed questionnaires were obtained from 364 maternity patients, with a response rate of 91%. Three hundred nine questionnaires were used for the analysis (55 questionnaires, or 15%, were ineligible due to exclusion criteria). Respondents completed the survey 1 to 4 days after giving birth, while still during hospitalisation at the maternity unit of the hospital participating in this study. At the same time, 50 questionnaires were sent to the internal information system’s office email to the staff of the obstetric unit (physicians and midwives) of the institution participating in the study. A total of 42 completed questionnaires were collected, with a response rate of 84%. Thirty-seven questionnaires were used for the analysis (5 questionnaires, or 9%, were ineligible due to exclusion criteria).

### Inclusion/exclusion criteria for the study respondents

The study included consenting maternity patients who had given birth (vaginally and by C-section) in the institution participating in this study. Due to factors that make the assessment more challenging and subjective, the study excluded mothers who were diagnosed with coronavirus during hospitalisation or inpatient care, who do not speak the national language and whose delivery resulted in a stillbirth (foetal demise in utero or during delivery). The inclusion criteria for the participating staff, much like the patients, included consent to participate in the study, as well as their job title (obstetrician-gynaecologist, midwife, resident doctors in obstetrics-gynaecology) and, most importantly, the nature of their work (at least part of the working time must be devoted to working in the maternity wards having direct contact with women in labour). Resident doctors in their first residency year and those who have completed their residency at another training site, as well as those who have completed less than 6 months of their residency in the last 12 months in the obstetric unit of the institution participating in the study, were excluded. The 6-month timeframe was selected as a minimum period necessary to understand the specifics of work and the typical midwifery practices within the institution.

### Research instrument

The questionnaire to assess the quality of obstetric care was developed by the study authors on the basis of one of the most commonly used questionnaires for assessing the quality of care in healthcare institutions, namely that by Ferguson et al,^13^ which examines the technical and functional aspects of service quality, external efficiency and the overall service quality rating. Although this questionnaire identifies 4 main blocks of questions and the components assessed by the questionnaire are universal, addressing the main links of the care delivery process, the specifics of midwifery and nuances of the clinical practice, some modification of the survey instrument were required. Many studies focussing on maternity patient satisfaction with the quality of care received in obstetric units have used the Intrapartal-Specific Quality from the Patient’s Perspective Questionnaire (QPP-I), structured by the Swedish researcher, prof. Bodil Wilde-Larsson, which is designed to assess the quality of care in labour and delivery.^14^ While this questionnaire addresses the main quality aspects that are of interest to the researchers (the assessment of staff competence, physical environment, person-centred approach, socio-cultural environment), the concepts of patient-centred care, value-based healthcare and respectful maternity care have been developing rapidly since 2010. Accordingly, instruments for measuring service quality satisfaction must also evolve. Moreover, given the disparities in healthcare systems, the specific clinical practice patterns of each country and institution, every country and healthcare organisation should develop their own instruments for assessing the quality of care.^15^ Therefore, while retaining the structure of the questionnaires used in healthcare quality research (based on the Ferguson questionnaire), and taking into account the aspects of quality to be assessed in the questionnaires (based on the QPP-1 questionnaire), the present study used a 50-question questionnaire for maternity patients and a 49-question questionnaire for medical staff, designed by the authors and focused on the most recent factors that determine and shape the quality of services. The questionnaires included an analysis of the technical quality indicators, functional quality indicators, evaluation of external efficiency and the analysis of the specific factors that affect the assessment.

The questionnaire given to the participants used a 5-point Likert scale to score the statements related to the aspects of the service quality assessment, where 0 is for ‘not evaluated/not applicable’, 1 is for ‘completely disagree’, 2 is for ‘partially agree’, 3 is for ‘agree’ and 4 is for ‘completely agree’. The internal reliability of the questionnaire scale was assessed by Cronbach’s alpha for each block of questions (Table 1).

**Table 1.** Cronbachs’ alpha coefficients for each block of questions.

Quantitative research questionnaire scales and subscales	Cronbach’s alpha
TECHNICAL QUALITY	.936
Environment of the maternity unit	.878
Arrangement of the premises	.858
Accessibility of services	.768
Staff qualification/competence	.976
FUNCTIONAL QUALITY	.950
Communication with healthcare providers	.950
Provision of information	.912
Participation in decision-making (patient empowerment)	.838
Staff empathy	.885
Communication with other medical staff	.759
SPECIFIC FACTORS AFFECTING THE ASSESSMENT	.704
EXTERNAL EFFICIENCY	.953

In all cases, Cronbach’s alpha was >.7, indicating good internal consistency. The overall internal reliability of the questionnaire is 0.966, indicating that the survey instrument is valid and appropriate for use. In order to test the comprehensibility of the questionnaire for the respondents participating in the study, a pilot study (10 forms) was conducted.

### Research ethics

The study has received permission from the institution’s Bioethics Committee to conduct a survey of patients and staff in the obstetric unit. The respondents were free to decide on their participation in the study. The anonymity of the respondents was preserved during the survey: no personally identifiable information was required and each respondent was assigned a unique number for data analysis. The survey questions were designed not to prejudice the respondents’ cultural, moral and religious beliefs.

### Methods of data analysis

Data were calculated using SPSS (Statistical Package for Social Sciences) software (version 20) and Microsoft Office Excel. Means (*M*) and standard deviations (SD) were calculated for the data expressed on an interval (Likert) scale, while frequencies in percentages (%) were calculated for the data expressed on a ranking scale. In all cases, a difference with a reliability greater than 95% (*P* < .05) was considered statistically significant.

## Results and Discussion

**Figure 1.**
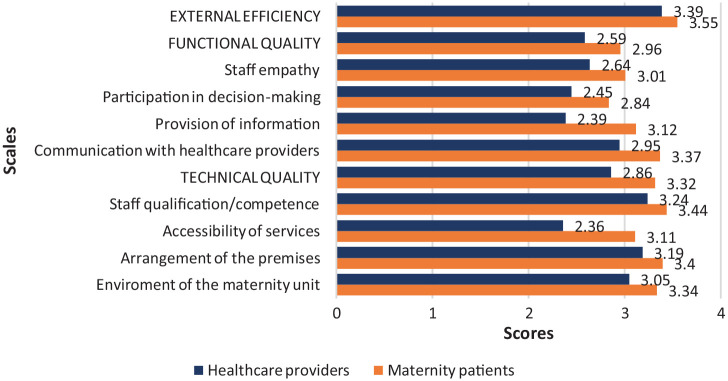
Comparison of service quality rating scales between the 2 study groups.

A comparative analysis of the attitudes of maternity patients and staff towards the quality of obstetric care demonstrated that maternity patients rated the quality of care higher than the medical staff in all quality rating scales studied (Figure 1). The greatest difference between the scores was observed for technical quality, whereas external efficiency scores differed the least between the staff and maternity patients. On the technical quality scale, the largest difference was observed in the accessibility of services, while on the functional quality scale, it was in the provision of information. These results suggest that, unlike in the study by Aniulienė et al,^7^ the staff, more than the patients, view not only the technical but also the functional quality of service more critically. Moreover, contrary to the findings of Gourevitch et al,^10^ the clinical community is equally focused on both quantitative and qualitative indicators, recognising their integrative importance for ensuring high-quality services. These claims are further illustrated by the comparative analysis of the assessment of the different quality aspects studied (Tables 2–4).

**Table 2.** Comparative analysis of the assessment of the technical quality statements.

	Maternity patients		Healthcare providers		*U*	*P*
	*M*	SD	*M*	SD		
TECHNICAL QUALITY	3.32	0.49	2.86	0.39	2790.5	.000
*Environment of the maternity unit*	3.34	0.52	3.05	0.51	3919.5	.001
The maternity unit has clear instructions for patients (signs) helping orienting themselves	3.39	0.62	2.86	0.86	3739.5	.000
Facilities enabling free movement in the maternity unit premises, depending on the condition are being provided (elevator, wheelchair, stairs)	3.48	0.51	3.35	0.63	5179.0	.284
The maternity unit is equipped with all the necessary treatment and care facilities	3.48	0.51	3.24	0.60	4582.0	.024
I am satisfied with the hospital food	3.01	0.73	2.33	0.59	1458.5	.000
*Arrangement of the premises*	3.40	0.53	3.19	0.56	4568.5	.030
Patients’ privacy is ensured (screens, curtains, separate space in the emergency department, maternity wards)	3.39	0.59	3.03	0.73	4168.5	.003
Examination equipment and the hospital premises is kept clean	3.40	0.54	3.35	0.54	5429.5	.565
*Accessibility of services*	3.11	0.62	2.36	0.62	2385.0	.000
I am satisfied with the waiting time in the emergency department (from arrival to examination)	2.98	0.74	2.46	0.61	3508.5	.000
There is enough staff in the department	3.23	0.70	2.27	0.84	2406.0	.000
*Staff qualification/competence*	3.44	0.52	3.24	0.48	4678.0	.039
The staff (doctors) perform their professional duties perfectly	3.44	0.52	3.16	0.50	4237.0	.003
The staff (midwives) perform their professional duties perfectly	3.43	0.52	3.32	0.53	5134.5	.243

Abbreviations: *M*, mean; SD, standard deviation; *U*, Mann-Whitney *U* test for differences between 2 independent groups; *P*, p value <0.05 considered statistically significant.

**Table 3.** Comparative analysis of the assessment of the functional quality statements.

	Maternity patients		Healthcare providers		*U*	*P*
	*M*	SD	*M*	SD		
FUNCTIONAL QUALITY	2.96	0.6	2.59	0.45	3150.0	.000
*Communication with healthcare providers*	3.37	0.48	2.95	0.42	2870.5	.000
Doctors introduced themselves during the first visit and were kind	3.41	0.52	2.92	0.60	3419.0	.000
Midwives introduced themselves during the first visit and were kind	3.42	0.53	3.00	0.58	3717.0	.000
Doctors were respectful	3.40	0.55	2.95	0.62	3633.0	.000
Midwives were respectful	3.41	0.54	3.16	0.60	4540.5	.019
Doctors listened to me and did not interrupt me when I spoke	3.34	0.60	2.67	0.68	2778.0	.000
Midwives listened to me and did not interrupt me when I spoke	3.36	0.56	2.92	0.55	3508.5	.000
The staff took into account the needs of the birth attendant during labour	3.34	0.49	2.92	0.64	3473.5	.000
The staff was quick to respond to my complaints and needs during labour	3.32	0.51	3.11	0.61	4695.5	.041
*Provision of information*	3.12	0.55	2.39	0.58	2061.5	.000
Doctors answered all my questions	3.07	0.72	2.76	0.68	4376.5	.012
Midwives answered all my questions	3.18	0.65	2.81	0.71	3995.0	.002
I was informed before each examination	3.36	0.57	2.41	0.72	1982.5	.000
I was provided with information about the procedures being made	3,19	0,69	2.38	0.89	2848.0	.000
I was informed about the steps taken during the procedures	3.27	0.61	2.05	0.91	1764.0	.000
I was provided with information about the results of the examinations and procedures made	3.04	0.70	2.46	0.69	3321.0	.000
I was informed about the possibility of giving birth in different labour positions	2.61	0.80	1.86	0.49	2249.0	.000
*Participation in decision-making (patient empowerment)*	2.84	0.63	2.45	0.51	3597.0	.000
I was able to take an active role in making decisions about my maternity care	3.17	0.62	2.64	0.76	3283.0	.000
I could choose the method of pain relief during labour	2.82	0.72	2.95	0.70	4537.5	.361
I could choose to give birth in my preferred labour position	2.62	0.78	1.97	0.55	2459.5	.000
It was possible to move freely during labour	2.52	0.87	2.27	0.80	3927.5	.070
*Staff empathy*	3.01	0.49	2.64	0.66	3539.5	.000
Doctors tried to understand my experience during labour	2.90	0.64	2.46	0.84	3759.5	.000
Midwives tried to understand my experience during labour	2.91	0.63	2.86	0.79	5442.5	.617
Doctors provided emotional support during labour	2.96	0.58	2.43	0.69	3253.0	.000
Midwives provided emotional support during labour	3.03	0.54	2.78	0.71	4511.0	.012

Abbreviations: *M*, mean; SD, standard deviation; *U*, Mann-Whitney *U* test for differences between 2 independent groups; *P*, p value  < .05 considered statiscally significant.

**Table 4.** Comparative analysis of the assessment of the external efficiency statements.

	Maternity patients		Healthcare providers		*U*	*P*
	*M*	SD	*M*	SD		
EXTERNAL EFFICIENCY	3.55	0.51	3.39	0.50	4422.0	.017
I am satisfied with the work quality of doctors in this maternity unit	3.59	0.51	3.31	0.67	4354.5	.013
I am satisfied with the work quality of midwives in this maternity unit	3.58	0.51	3.50	0.56	5183.5	.436
I am satisfied with the services in this maternity unit	3.61	0.52	3.44	0.56	4695.5	.077
The quality of services in this maternity unit is high	3.66	0.49	3.54	0.56	5096.5	.190
I would recommend this maternity unit to my relatives/friends	3.46	0.66	3.14	0.67	4215.5	.004

Abbreviations: *M*, mean; SD, standard deviation; *U*, Mann-Whitney *U* test for differences between 2 independent groups; *P*, p value  < .05 considered statiscally significant.

Statistically significant differences (*P* < .05) were found for most of the statements on the technical quality scale, indicating that the maternity patients rated the environment of the healthcare institution, its facilities and the accessibility of services significantly higher than the staff providing the services (Table 2).

Given that staff can assess the technical quality situation more objectively because they have an insider’s knowledge of the issues, have spent a lot of time in the institution and have a certain level of rapport with the patients and knowledge of their experiences, these results are not surprising. Meanwhile, the results on the functional quality scale, when compared with other studies, showed statistically significant differences, suggesting that (much like for the technical quality scale) maternity patients rated individual aspects of functional quality for the majority of the statements significantly higher than the staff (Table 3).

Both physicians and midwives are therefore not only aware of the existing/potential gaps in functional quality, but also view them more critically than the mothers themselves, who, due to their own specific condition during the in-patient care period (physical pain, uncertainty, stress, anxiety and other emotions associated with childbirth), are expected to be rather ‘strict’ in their own assessment of the services they receive. Furthermore, it seems that physicians are aware of the importance of empathy and effective communication, and, like patients, share a holistic approach to the delivery of care.^10^ This in turn should reduce the volume of quality improvement interventions and further implies that the medical staff are interested in delivering high-quality services.

It is worth mentioning that the results of this study highlighted that when it comes to the functional quality aspects (especially information provision, empowerment of the patient to participate in the decision-making process), both the maternity patients and healthcare providers rated the services provided by midwives higher than those of physicians (Table 3) although on some aspects the difference on the patient side seems to be relatively small. For example, referring to staff empathy the mean difference is 2.90 versus 2.91 and 2.96 versus 3.03 regarding quality of services provided by midwives and doctors so further research on different aspects of services provided by these healthcare specialists might give more accurate picture on the situation in maternity units as results obtained by this research were statistically insignificant. Nevertheless, taking into account the importance of patient’s perspective evaluating service quality such findings implement the need to analyse differences in service quality provided by midwives and doctors in other obstetrical units throughout the country in order to make more reliable conclusions.

These statements, designed to prompt the 2 groups of participants to compare the quality of care provided by midwives and physicians, had the primary purpose of determining whether midwives adequately respond to the needs of the maternity patients. This comparison serves to justify the need for a model of midwife-only delivery care for a normal, risk-free birth. The number of midwife-assisted deliveries is gradually increasing in tertiary healthcare facilities, but this practice is much rarer in secondary care providing facilities as mentioned earlier in the article, where the number of normal deliveries is higher but the physician is usually more or less involved in all deliveries and all stages of labour. By contrast, although this model of delivery care is not yet widespread enough in European countries, there is a growing body of evidence in the scientific literature that supports favourable outcomes in deliveries supervised by midwives alone, with fewer interventions and, most importantly, higher patient satisfaction with the quality of care. In Switzerland, where women are offered midwife-assisted birth in the absence of pregnancy and childbirth risks, a study found that midwife-assisted care is a safe alternative to deliveries supervised by a physician, with a lower rate of C-sections among midwife-attended births.^16^ A systematic review by Rayment^17^ aimed at better defining the standards of midwifery delivery care, states that, although the availability of this type of service (author’s note: childbirth care provided by a midwife) is rarely applied in most parts of Europe, it offers excellent clinical outcomes, lower incidence of obstetric interventions and a high perceived quality of such services. A higher rate of adverse outcomes in midwife-attended births was also not observed by Martin-Arribas et al,^18^ who compared delivery outcomes in cases attended by physicians to those attended by midwives in a population of Spanish mothers. With births supervised by a midwife, it must, of course, be clearly established when a change in the clinical situation requires physician’s involvement. It should also be stressed that this model of care requires quite substantial organisational changes, first of all in the training of midwives and in terms of increasing the appeal of this medical speciality as well as the respect for midwife profession. This is especially applicable for hospitals that are more removed from large urban centres and experience a shortage of midwives or do not have resident doctors who can, during their residency, take over certain part of the services provided by midwives and where the accessibility of services in general is a serious problem. However, from an economic point of view, midwives require less training at a lower financial cost than obstetricians and gynaecologists. A study in the United States confirmed the economic benefits of midwife-attended births, finding an increase in financial efficiency with a lower rate of obstetric interventions such as episiotomies under midwife supervision.^19^ Therefore, increasing the number of employed midwives should be a priority area for improving the quality of maternity care, not least because, according to the current wording of the medical standard of midwifery in Lithuania, midwives have the authority to ‘supervise the maternity patient during all periods of spontaneous childbirth in the absence of high-risk factors, to independently assist with a spontaneous delivery when there are no high-risk factors, and to deliver a breech birth in emergency situations’.^6^

As mentioned above, the external efficiency scale showed the least difference of opinion in terms of the statements given to the respondents. However, compared to the healthcare providers, maternity patients were statistically significantly more satisfied with the physician’s work and more likely to recommend the institution participating in the study to their relatives and friends (Table 4).

## Conclusions

1. A comparative analysis of the assessment of the quality aspects from the perspective of maternity patients and healthcare providers demonstrated that the most significant difference between their views was in the assessment of the technical quality, while the least significant difference was in the assessment of the external efficiency, which was scored high by both groups of the respondents – the healthcare institution participating in this study provides high-quality services.
2. The maternity patients rated healthcare services provided higher than the staff across all the scales and for most of the statements, and this difference was statistically significant, which suggests that, especially in midwifery, the staff approach quality assurance as a complex process and they recognise the importance of all aspects of quality assurance, rather than focussing on quantitative indicators alone. This in turn indicates an openness to the process of quality improvement and to the possibility of implementing appropriate changes.
3. Only a comprehensive assessment of the quality assurance aspects as regarded by the patients and the staff (including not only specialised physicians, but also resident doctors and, for obstetric services, midwives) can provide truly comprehensive and objective information on the quality assurance of the service delivery process therefore should be included in the regular quality assessments of healthcare facilities as one of the most informative assessment tools.
4. Both maternity patients and healthcare providers, albeit by a minimal difference, rated the services provided by midwives higher than those provided by physicians on the scales of staff communication, information provision and staff empathy. Therefore, it is appropriate to increase the appeal of midwifery, the number of midwifery professionals, to ensure growing number of midwife-only deliveries in low-risk births. This would in turn reduce the risk of professional burnout and the workload of physicians, especially given that the care of normal childbirth in all its stages is within the scope of competence of the midwife in Lithuania, in accordance with the current wording of the medical standard of midwifery.
